# Nonalcoholic Fatty Liver Disease in South Asia

**DOI:** 10.5005/jp-journals-10018-1189

**Published:** 2016-12-01

**Authors:** Girish K Pati, Shivaram P Singh

**Affiliations:** 1 Department of Gastroenterology, Shri Ramachandra Bhanj Medical College, Cuttack, Odisha, India; 2 Kalinga Gastroenterology Foundation, Cuttack, Odisha, India

**Keywords:** Fatty liver, Hepatocellular carcinoma, Nonalcoholic fatty liver disease, nonalcoholic steatohepatitis, Physical activity, South Asia.

## Abstract

Nonalcoholic fatty liver disease (NAFLD) is the most common chronic liver disease in the West, and is also increasing alarmingly in South Asia, reaching an epidemic proportion of 30% because of epidemic of obesity and metabolic syndrome (MS) in younger South Asians in the last two decades. Prevalence of MS and fatty liver is escalating in geometric progression in South Asian countries, such as India, Pakistan, Sri Lanka, Bangladesh, Nepal, Bhutan, Burma, and Maldives because of sedentary lifestyle, poor health awareness, socioeconomic growth, affluence, urbanization, and dietary westernization. Almost 20% of world’s population resides in South Asia, making it the most populous and most densely populated geographic region in the world, thereby having most of MS and NAFLD cases within its territory. The risk factors and course of NAFLD do not differ between South Asians and other ethnic populations, but the obesity epidemic is more recent in South Asia than elsewhere in the world. Nonalcoholic fatty liver disease may progress through stages of simple bland steatosis, nonalcoholic steatohepatitis (NASH), hepatic fibrosis, cirrhosis, and finally hepatocellular carcinoma (HCC). It is frequently associated with obesity, MS, dyslipidemia, insulin resistance (IR), and type-2 diabetes mellitus (DM). Nonalcoholic fatty liver disease is frequently diagnosed with abdominal ultrasonography (US) study. Despite its high prevalence in the community till now, no definitive pharmacotherapy is available for NAFLD. However, modification of risk factors, such as dyslipidemia, control of diabetes, and weight reduction do help to some extent. The nonobese South Asians are also at increased risk of having NAFLD and NASH as, despite of absence of frank obesity in South Asians, they are metabolically more obese compared to other ethnic population and more prone to develop NAFLD-related complications. Therefore, the cost-effective US abdomen should be included in the list of tests for persons undergoing preemployment or master health checkups for early diagnosis of NAFLD in this resource-constraint South Asian region, so that early necessary measures can be undertaken to reduce NAFLD associated morbidity and mortality in the community.

**How to cite this article:**

Pati GK, Singh SP. Nonalcoholic Fatty Liver Disease in South Asia. Euroasian J Hepato-Gastroenterol 2016;6(2):154-162.

## INTRODUCTION

Nonalcoholic fatty liver disease (NAFLD) is a clinico-histopathological entity in which liver histology resembles alcohol-induced liver injury, which occurs in patients who never drink or drink less alcohol (< 20 g/day in male; < 10 g/day in female).^1^ Fatty liver by definition means fatty infiltration of at least 5% of the liver tissue.^2^ Nonalcoholic fatty liver disease cases may progress through stages of simple bland steatosis, nonalcoholic steatohepatitis (NASH), hepatic fibrosis, cirrhosis, and finally hepatocellular carcinoma (HCC). Patients with NASH have most of the component of metabolic syndrome (MS), and prone to suffer from cardiac, cerebrovascular diseases, diabetes-related complications, and liver-related morbidities and mortality.^3^ Prior report suggests that 80% of cryptogenic cirrhostics suffer from NASH.^4^ In the current era, NAFLD is considered to be the most common liver disease in the western countries, affecting 20 to 30% of the general population^5,6^ and is also increasing alarmingly in South Asian countries, with an epidemic proportion of 30% due to epidemics of obesity and MS in the younger South Asians in the last two decades.^7^ Prevalence of MS and fatty liver is escalating in geometric progression in South Asian countries, especially India, Pakistan, Sri Lanka, Bangladesh, and Nepal because of sedentary lifestyle, poor health awareness, socioeconomic growth, affluence, urbanization, and dietary westernization. Almost 20% of the world’s population resides in South Asia, making it the most populous and most densely populated geographic region in the world, and South Asia harbors a large number of MS and NAFLD cases within its territory. The risk factors and course of NAFLD probably do not differ between South Asians and other ethnic populations, but the recent obesity epidemic in South Asia may lead to increased occurrence of fatty liver in this part of the world. Earlier studies have confirmed that the South Asians are at higher risk to suffer from obesity-related morbidity and mortality compared to other ethnic groups, including the Caucasians.^8^ Nonalcoholic fatty liver disease is considered to be the hepatic manifestation of MS, and commonly associated with insulin resistance (IR) and other components of MS, such as diabetes mellitus (DM), hypertension, central obesity, hypertriglyceridemia, and low high-density lipoprotein (HDL) cholesterol.^9^ Nonalcoholic fatty liver disease is among the most common etiologies of unexplained transaminitis, in particular raised alanine aminotransferase (ALT) has been adopted as a surrogate marker of NAFLD in epidemiological studies.^10^ The prevalence of NAFLD in the West,^11^ Asia,^9,12^ type-2 DM patients,^13^ and obese population^14^ varies from 24 to 42, 5 to 40, 50 to 75, and 35 to 75% respectively. Men out-numbered the females in having NAFLD in most of the published series.^11,15^ Pathogenesis of NAFLD involves a multihit hypothesis; initially hepatic steatosis occurs due to IR, followed by hepatocellular inflammation due to oxidative stress.^16^ Current report suggests that altered gut microbiome may be associated with higher endotoxemia and lower cecal bifidobacterium contributing to diabetes, obesity, and NASH.^17^ Most patients with NAFLD are usually asymptomatic, or may present with fatigability, heaviness, and discomfort on the right side of the upper abdomen.^18^ Nonalcoholic fatty liver disease is usually diagnosed by abdominal ultrasonography (US) imaging. The ultrasound features of NAFLD include increased hepatic echogenicity, vascular blurring, and deep attenuation of US signals. These US features had good accuracy in detecting fatty liver and had good correlation with visceral obesity and MS.^19,20^ Magnetic resonance imaging (MRI) can quantify the triglycerides stores in liver, which may be useful in assessing the efficacy of therapeutic intervention.^21^ For assessment of disease severity, liver histology study is required, which can clearly differentiate NAFLD from NASH and liver fibrosis, which is never possible by other available imaging modalities. Despite its high prevalence in the community till now, no definitive pharmacotherapy is available for NAFLD. However, modification of risk factors, such as dyslipidemia, control of diabetes, and weight reduction do help to some extent.^22^ Lifestyle changes and weight loss remain the mainstay of therapy, and are effective in improving liver function tests and histology. Treatment strategies for NAFLD have resolved around identification and treatment of frequent associated metabolic conditions, such as diabetes, obesity, dyslipidemia, hypertension, improving IR by weight loss, exercise, and/or pharmacotherapy, and by using hepatoprotective drugs, such as antioxidants, vitamin E, high-dose ursodeoxycholic acid to protect liver from secondary insults.^23,24^ Prior study suggested an emerging role for bariatric surgery, resulting in both biochemical and histological improvements in patients with NASH.^25^ Bariatric surgery can cure diabetes in ~66%, and reverse NASH in ~80% of cases; whereas effect on fibrosis is less clear.^26^ Compared to West, in South Asian countries including India, many cases suffer from acute viral hepatitis A and E and chronic viral hepatitis B and C because of densely populated regions in this part of the world, except two South Asian countries, such as Pakistan and Nepal, where chronic viral hepatitis B almost eradicated. Whenever cases with NAFLD also get infection from acute or chronic viral hepatitis infection, they rapidly deteriorate and may land in acute or chronic liver failure and increased morbidities and mortalities. As this South Asian countries are resource-constrained poor developing countries where a lot of people are illiterate and do not adopt safety lifestyle measure, so they are prone to suffer from these potential viral infections and poor outcome. Therefore, it becomes imperative that the primary physicians in the South Asian countries should be well aware regarding these potential fatal liver diseases and hepatotrophic viral infection in this region of the world so that they can well guide their patients to adopt healthy preventive lifestyle measures and that they will suffer less commonly from these preventable liver-related diseases.

## EPIDEMIOLOGY AND DETERMINANTS

The initial astute observation of relationship between central obesity and IR with high prevalence of diabetes and cardiovascular risk in South Asians compelled the attention of whole world on the high prevalence of MS in this particular region.^27^ Studies from India^28,29^ and Sri Lanka^30,31^ have led authors to conclude that the prevalence of NAFLD in South Asia varies from 9 to 45%. The lowest prevalence (8.7–18%) of NAFLD was observed in physically active, poor, lean persons residing in rural region.^29,31^ Prior report suggested that the presence of central obesity, visceral fat, and cardiovascular risk factors is higher in South Asians compared to Caucasians with similar body mass index (BMI) and lower average waist circumference (WC).^32^ For equivalent levels of overnutrition, South Asians are more prone to MS, type-2 DM, and NAFLD than Europeans because of differences in body composition, particularly adiposity and muscle bulk. Lifestyle changes are likely to account for the pandemics of fatty liver, obesity, and type-2 diabetes in South Asia. Though South Asians are not more overt obesity compared to other ethnic populations, but they were metabolically more obese compared to other ethnic groups.^33^ Clustering of cardiovascular risk factors in South Asians was first reported from UK.^34^ South Asians usually have higher percentage of visceral body fat,^35,36^ abdominal obesity,^35,36^ IR,^37^ hyperinsulinemia,^38^ and low muscle mass^39^ compared to other ethnic populations; therefore they are more prone to suffer from NAFLD and MS. Studies in North America have noted an ethnic predisposition for occurrence of NAFLD in South Asian Indian males, Hispanics, and East Asians, despite similarities in metabolic risk factors between different races.^40,41^ In particular, central obesity/truncal obesity is much more common in South Asians and also evident in nonobese South Asians. Further, thick subcutaneous adipose tissue in South Asians may be a key correlate of IR.^42,43^ South Asians usually have thick subscapular subcutaneous fat since birth, therefore commonly associated with hyperinsulinemia and suffer from NAFLD and MS at an early age compared to other ethnic population.^44^ Genetic propensity for development of dyslipidemia, obesity, and diabetes has been observed in South Asians.^45-48^ Various studies showed that South Asians had increased prevalence of hyperglycemia, dyslipidemia, IR, procoagulant activity, and large adipocytes.^49-51^ Other causative factors for development of MS and NAFLD in South Asians are less physical activity, spending much time watching televisions and computer games, increased consumption of energy-rich imbalanced food, junk food, soft drink, fast food compared to other ethnic population.^49,52,53^ South Asians usually consume comparatively lesser amount of omega-3 polyunsaturated fatty acids (PUFA), monosaturated fatty acids, and more amount of omega-6 polyunsaturated fatty acids compared to British Caucasians.^54^ Prior study confirmed the relationship of NASH with low serum adiponectine levels in Asians.^11^ Schwimmer et al^55^ suggested that familial factors might act as a major risk factor for occurrence of NAFLD in South Asians. All these abovementioned factors and determinants play significant role in increased prevalence of MS, NAFLD, type-2 DM, and cardiovascular morbidity and mortality in South Asians. In most Western studies, the mean age of presentation of NAFLD patients was 50 to 55 years,^1,10,56^ which was higher compared to NAFLD cases in South Asian regions, with mean age of presentation at 45 years in Pakistan,^57^ and 38 years in India.^58^ Mean BMI of Western population^1,56^ with NAFLD was 30 to 35 kg/m^2^, which was in sharp contrast to South Asians with NAFLD, with mean BMI of 29 kg/m^2^ in India^58,59^ and 27 kg/m^2^ in Sri Lanka.^30^ Presence of excess dorsocervical fat and excess fat deposition under the skin (double chin) signify heightened risk for development of MS in South Asian Indians and can be used as a phenotypic marker.^59^ South Asian Indians are more prone to develop IR, MS, type-2 DM, and coronary heart disease because of presence of higher body fat, abdominal (central) adiposity, and higher high-sensitivity C-reactive protein (hs-CRP) levels compared to white Caucasians.^60,61^ Study by Babusik et al^62^ at Kuwait reported that in South Asians, increased age, male gender, hyperglycemia, increased WC, and waist height ratio were significantly associated with hepatic steatosis.

## PREVALENCE AND ASSOCIATION IN DIFFERENT SOUTH ASIAN COUNTRY

### India

Prevalence of NAFLD in India would approximate prevalence of MS since most of metabolic covariates of NAFLD are highly prevalent in Indians.^63^ Prior study indicated that the prevalence of MS in India is 11 to 41%.^63,64^ The community prevalence of NAFLD in India varies from 5 to 28%.^65-67^ Almost 30 to 65% of adult urban Indians are either overweight/obese or have abdominal obesity.^60^ Common age of presentation of NAFLD in Indians is 30 to 50 years.^68^ Diabetes and central obesity are common predisposing factors, while IR is detected almost universally.^65-67,69^ A study conducted by Bajaj et al,^70^ at New Delhi, reported that subjects with NAFLD had significantly higher degree of BMI, WC and hip circumference, fasting hyperglycemia, fasting hyperinsulinemia, hypercholesterolemia, and hypertriglyceridemia. A study by Nigam et al,^71^ at New Delhi, demonstrated that presence of higher BMI, high-sensitive C-reactive protein (hs-CRP) and WC, and fasting hyperglycemia, hypercholesterolemia, hypertriglyceridemia, hypertension, and MS were significantly and independently associated with NAFLD compared to controls. One recent study by Singh et al^72^ at Cuttack, Odisha revealed that Indians with NAFLD were younger, had lower BMI, and prevalence of DM, MS but similar necroinflammatory activity and fibrosis score compared to the West. Transaminitis is neither a reliable marker of NASH nor fibrosis and should not influence the decision for liver biopsy in these patients.^72^ The common primary diseases for which these patients sought consultation were nonulcer dyspepsia (54.5%) and irritable bowel syndrome (29.4%).^72^ A study by Kumar et al^73^ in India reported that the lean NAFLD cases were less commonly diabetic (p = 0.01) and had significantly lesser degree of fasting hyperinsulinemia, homeostasis model assessment insulin resistance (HOMA-IR), and MS (p < 0.001) compared to obese NAFLD cases. The serum lipid profile was almost similar between the obese and lean NAFLD cases and 89% of lean NAFLD cases had dyslipidemia. The lean NAFLD cases had significantly lesser degree of hepatic necroinflammatory activity (p = 0.05) and mild-to-moderate fibrosis (p < 0.001), despite similar incidence of dyslipidemia, steatohepatitis, and advanced fibrosis compared to obese cases. A recently published study by Singh et al^74^ in coastal eastern India reported that the prevalence of diabetes and prediabetes was six times more common in NAFLD patients compared to healthy controls. Moreover, NAFLD patients with diabetes had higher metabolic risk factors, such as large waistline, hypertension, high triglyceride levels, and increased IR. Diabetes or prediabetes patients per se do not have histologically severe disease, rather IR plays an important role in increasing the severity of the disease. Another study by Singh et al^75^ in coastal Eastern India stated that nearly half of NAFLD patients in this coastal part had no IR; one-third of them had significant fibrosis, and therefore, the author opined that NAFLD might be a heterogeneous disease and sole IR might not be the sole etiologic factor for progression of NAFLD; rather some unknown factors might play role in increasing severity of NAFLD cases.

### Sri Lanka

Studies from Sri Lanka have reported a NAFLD prevalence of 32.6% in an urban community^30^ and 18% in a predominantly Indian Tamil, rural, physically active, economically deprived estate worker community.^31^ Obesity, acanthosis nigricans, fasting hyperglycemia, IR, hypertension, hypertriglyceridemia, and transaminitis were independently associated with NAFLD.^30^ Male sex, high BMI, high WC, hypertension, and hyperglycemia were significantly associated with NAFLD.^31^ Sri Lankan studies have shown that the risk of developing diabetes within 3 years of NAFLD diagnosis increases 3 to 4 fold, even among lean individuals. A person with NAFLD was 1.6 times more likely to develop DM compared to a person without NAFLD.^30^ A study by De Hewavisenthi et al^76^ showed that 35.1% of study population had NASH, with majority (79%) cases observed in male and NASH was associated with increased prevalence of DM (55%), obesity (52%), hyperlipidemia (54%), family history of risk factors (66%), and consumption of high dietary fat intake (66%).

### Pakistan

The prevalence of NAFLD in rural and urban areas in Pakistan among lower, middle, and higher societies were 9, 15, 27%, and 21, 27, 42% respectively reflecting the effects of industrialization and urbanization on higher prevalence of fatty liver in urban higher societies.^77^ The prevalence of NAFLD in patients with type-2 DM and MS in Pakistani population was found to be 72.4%.^78^ A study by Niaz et al^79^ showed that 13.5% of study population had elevated ALT level of unknown etiology and found to have NAFLD. Previous studies reported that 66.6^79^ and 49.5%^80^ cases with transaminitis had NAFLD. A study by Khurram and Ashraf^57^ revealed that presence of obesity, hepatomegaly, diabetes, and hypertriglyceridemia was characteristically associated with higher prevalence of NAFLD in community with majority of cases found in females. All the NAFLD cases presented with fatigability and 38% cases had NASH. A study by Abbas et al^81^ reported that cases with NAFLD were significantly older and had significantly higher BMI compared to controls. 21.8% cases with NAFLD had significantly raised ALT level and presence of NASH.^81^ A study by Luxmi et al^82^ reported that the prevalence of fatty liver in type-2 diabetics was 60.8%, which was similar to that reported by Gupte et al^13^ in India (NAFLD prevalence in diabetics was 49%). High BMI was found as an independent predictor of fatty liver.^82^ A study by Taseer et al^83^ reported that the prevalence of NAFLD in the diabetic patients was 51% and majority (92.15%) of patients with NAFLD had hypertriglyceridemia and most common presentation of NAFLD was heaviness in the right upper abdomen (64.7%).

### Bangladesh

A study by Khan et al^84^ reported that the prevalence of NAFLD was 44%, with majority (54%) of cases found in male. Majority of cases (59.3%) presented at the age of 40 to 60 years and MS was present in 61.5% of cases.^84^ A study by Alam et al,^85^ the largest study on NAFLD from Bangladesh, reported that common age of presentation of NAFLD was 30 to 50 years. Most cases were females and majority (96.2%) cases had central obesity. Prevalence of NASH was 42.4%^85^ in NAFLD cases, which is much higher compared to other published report.^11^ Presence of diabetes and high serum GGT could significantly predict presence of NASH.^85^

### Nepal

A study by Mittal et al^86^ at Pokhara, Nepal reported that the prevalence of NAFLD was 17%. Mild-to-moderate elevations in serum levels of aspartate aminotransferase (43.42–49.49 IU/L) and ALT (43.90–53.92 IU/L) were the most common laboratory abnormalities found in patients with NAFLD.

Prevalence, mean age of presentation, sex predominance, and associations with different metabolic parameters of NAFLD cases residing in South Asia are described in Tables 1 to 6.^87^-^95^

**Table 1:** **Table 1:** Prevalence of NAFLD in South Asia (India)

*Country (region)*		*Population category*		*Mode of diagnosis*		*No. of subjects (n)*		*Prevalence (%)*		*Authors*		*References*	
East India		Asymptomatic healthy attendants		US study		159		24.5		Singh et al		87	
North India		Hospital patients		US study		2,156		4		Anand et al		88	
West India		General population		US study		1,168		16.6		Amarapurkar et al		68	
East India		Gastroenterology patients		US study		639		21.6		Singh et al		89	
South India		Urban general population		US study		541		32		Mohan et al		28	
West India		Routine health checkup		US study		1,003		22.6		Uchil et al		90	
North India		Laparotomy patients		Histology		57		42		Agrawal et al		91	
North India		General autopsy		Histology		100		40		Bal et al		92	
East India		Road traffic accident victims		Histology		103		14.6		Singh et al		93	
North India		Healthy volunteers		US study		121		32		Bajaj et al		70	

**Table 2:** **Table 2:** Prevalence of NAFLD in other South Asian countries

*Country (region)*		*Population category*		*Mode of diagnosis*		*Number of subjects (n)*		*Prevalence (%)*		*Authors*		*References*	
Pakistan (Rawalpindi)		Ex-army personnel and their dependents		US study		207		50		Bano et al		80	
Pakistan (Karachi)		Visitors attending hepatitis awareness program		US study		928		15.3		Abbas et al		81	
Pakistan (Karachi)		Routine health checkup		US study		952		13.5		Niaz et al		79	
Sri Lanka		Asymptomatic transamnitis		Histology		296		35.1		De Hewavisenthi et al		76	
Sri Lanka		Urban general population		US study		2,985		32.6		Dassanayake et al		30	
Sri Lanka (Nuwara Eliya)		Rural physically active population		US study		403		18		Pinidiyapathirage et al		31	
Bangladesh		Hospital-based study population		US study		334		44		Khan et al		84	
Nepal (Pokhara)		Hospital-based study population		US study		515		17		Mittal et al		86	

**Table 3:** **Table 3:** Mean age of presentation and sex predominance among South Asian (Indian) NAFLD patients

*Country (regions)*		*Numbers of patients (n)*		*Sex predominance*		*Mean age of presentation (years)*		*Authors*		*References*	
East India		63		Male		42.7		Singh et al		89	
West India		730		No sex predominance		39.8		Amarapurkar et al		68	
West India		225		Male		Nm		Uchil et al		90	
North India		100		Male		37.8		Duseja et al		58	
North India		150		Male		42.2		Bhat et al		94	
East India		336		Male		41.7		Singh et al		75	
East India		515		Male		46.6 (Diabetic)		Singh et al		74	
						40.4 (Nondiabetic)					
East India		632		Male		42.4		Singh et al		72	
North India		205		Male		38 (Lean)		Kumar et al		73	
						40.9 (Obese)					

Nm: Not mentioned

**Table 4:** **Table 4:** Mean age of presentation and sex predominance in other South Asian countries

*Country (regions)*		*Numbers of patients (n)*		*Sex predominance*		*Mean age of presentation (years)*		*Authors*		*References*	
Pakistan (Rawalpindi)		103		Female		45.3		Bano et al		80	
Pakistan (Karachi)		128		Nm		39.2		Niaz et al		79	
Pakistan (Rawalpindi)		50		Female		39.12		Khurram and Ashraf		57	
Pakistan (Karachi)		142		Male		43.3		Abbas et al		81	
Sri Lanka		103		Male		37.2		De Hewavisenthi et al		76	
Sri Lanka		973		Female		52.8		Dassanayake et al		30	
Sri Lanka (Nuwara Eliya)		73		Male		50.5		Pinidiyapathirage et al		31	
Bangladesh		146		Male		Nm		Khan et al		84	
Bangladesh		439		Female		40.8		Alam et al		85	

Nm: Not mentioned

**Table 5:** **Table 5:** Nonalcoholic fatty liver disease associations in South Asia (India)

*Region*		*Number of cases (n)*		*BMI in kg/m^2^ (mean)*		*Obesity (%)*		*Dysglycemia (%)*		*MS (%)*		*Authors*		*References*	
Allahabad		39		26.7		66.7		23.1 (IFG)		41		Bajaj et al		70	
Mumbai		226		28.5		24.8		72.4 (IFG)		47.1		Uchil et al		90	
Chandigarh		100		Nm		68		12 (DM)		50		Duseja et al		58	
New Delhi		51		26.7		69.4		10 (DM)		20.9		Madan et al		15	
Odisha		39		25.9		12.8		41.7 (IR)		Nm		Singh et al		87	
Chandigarh		127		28.7		68		83 (IR)		48		Duseja et al		95	
								13 (DM)							
Odisha		336		26.3		68.7		54.4 (IR)		31.6		Singh et al		75	
								19.9 (DM)							
Odisha		515		26.3 (Nondiabetic)		Nm		22.9 (Prediabetic)		Nm		Singh et al		74	
				26.6 (Diabetic)				24 (DM)							
Odisha		632		26.1		61.7		15.2 (DM)		40		Singh et al		72	
								54.4 (IR)							
New Delhi		205		21.3 (Lean)		68.7		3.7 (Lean diabetic)		22 (Lean)		Kumar et al		73	
				28.3 (Obese)				7.4 (Lean IR)		64 (Obese)					
								26 (Obese diabetic)							
								61 (Obese IR)							

BMI: Body mass index; IFG: Impaired fasting glucose; MS: Metabolic syndrome; IR: Insulin resistance; DM: Diabetes mellitus; Nm: Not mentioned

**Table 6:** **Table 6:** Nonalcoholic fatty liver disease association in other South Asian countries

*Country (location)*		*Case (n)*		*BMI kg/m^2^ (mean)*		*Obesity (%)*		*Dysglycemia (%)*		*MS (%)*		*Authors*		*References*	
Pakistan (Rawalpindi)		103		31.3 (F)		66		34 (DM)		28		Bano et al		80	
				27.7 (M)											
Pakistan (Rawalpindi)		50		32.6		54		30 (DM)		Nm		Khurram and Ashraf		57	
Pakistan (Karachi)		142		28.6		77.46		9.85 (DM)		Nm		Abbas et al		81	
Pakistan (Karachi)		128		27.1		Nm		Nm		Nm		Niaz et al		79	
Sri Lanka		103		Nm		52		55 (DM)		Nm		De Hewavisenthi et al		76	
Sri Lanka		973		27.1		69		66.9 (IFG)		Nm		Dassanayake et al		30	
Sri Lanka (Nuwara Eliya)		73		Nm		Nm		43.5 (IR)		Nm		Pinidiyapathirage et al		31	
Bangladesh		146		Nm		Nm		Nm		61.5		Khan et al		84	
Bangladesh		439		Nm		75.1		16.8 (DM)		42.9		Alam et al		85	

N: Number; M: Male; F: Female; MS: Metabolic syndrome; IFG: Impaired fasting glucose; DM: Diabetes mellitus; IR: Insulin resistance; Nm: Not mentioned

## CONCLUSION

Nonalcoholic fatty liver disease represents only the tip of iceberg, what we see, whereas it reflects the ongoing devastating process inside the body, i.e., occurrence of MS or increased risk for its occurrence in body with time if not properly managed at an early stage, which is essentially a preventable condition. This review highlights the fact that the South Asians are at increased risk of having NAFLD and NASH and these should be searched even in nonobese individuals, as, despite absence of frank obesity in South Asians, compared to other ethnic population, they are relatively more metabolically obese and more prone to develop NAFLD and related complications compared to other ethnic populations. Therefore, the cost-effective US of abdomen should be included in the list of tests for persons undergoing health or preemployment checkups for early diagnosis of NAFLD in this resource-constraint South Asian region, so that early necessary measures can be undertaken to reduce NAFLD associated morbidity and mortality in the community. Whether residing in the East or West, the South Asians seem to be at very high risk to suffer from fatty liver compared to Caucasians. In the West, patients with NAFLD were usually old, females, and obese, whereas South Asians with NAFLD were usually young, males, and nonobese. Nonalcoholic fatty liver disease is likely to soon become the largest contributor of liver-related morbidity and mortality like it is in the West. Pharmacological therapy for NAFLD is still evolving and large-scale primary prevention health education strategy and lifestyle programs are required to stem this tide, while further research is to be done to identify missing links in the pathogenesis and treatment of NAFLD in future.
